# Involvement of a novel circularRNA, hsa_circ_0000520, attenuates tumorigenesis of cervical cancer cell through competitively binding with miR‐146b‐3p

**DOI:** 10.1111/jcmm.15414

**Published:** 2020-06-27

**Authors:** Jinling Zhang, Ruyu Cai, Yifan Zhang, Xiaoyu Wang

**Affiliations:** ^1^ Department of Gynaecology Shen Zhen People’s Hospital the Second Clinical Medical College of Jinan University Shenzhen China; ^2^ Department of Gynaecology the First Affiliated Hospital of Jinan University Guangzhou China

**Keywords:** cervical cancer, circRNAs microarray, hsa_circ_0000520, miR‐146b‐3p, PAX5

## Abstract

The implication of circular RNAs (circRNAs) in the pathogenesis of human cervical cancer (CC) has been demonstrated by numerous of researches, nevertheless, the whole regulatory network of circRNAs in CC remains unclear. In the present study, two GSE data sets (GSE113696 and GSE102686) were enrolled to analysed different expressed circRNA. We found that hsa_circ_0000520(circ_0000520) was decreased in CC tissues and cell lines. Functional studies indicated circ_0000520 overexpression in vitro repressed CC cell proliferation, invasion and migration, while promoted CC cell apoptosis. Moreover, circ_0000520 overexpression in vivo repressed CC tumour growth. Mechanismly, circ_0000520 and PAX5 were revealed to directly bind to miR‐146b‐3p, and circ_0000520 could indirectly regulate PAX5 by sponging miR‐146b‐3p. In conclusion, **c**irc_0000520 repressed CC progression in vitro and in vivo by sponging miR‐146b‐3p to release PAX5.

## 1 INTRODUCTION

Cervical cancer (CC) is a kind of human cancer that occurs in cervix, and it developed as the second most frequent malignancy in females in recent years.^1^ According to the estimation of the American Cancer Society, 13 170 new CC cases will be diagnosed, and approximately 4250 deaths will occur in the United State in 2019 alone.^2^ Infection of human papillomavirus (HPV) is one of the primary reasons that lead to CC.^3^ Although the world incidence and mortality of CC has been significantly improved since the development and application of HPV vaccine, there still have numerous populations developed into CC.^4^ The five‐year survival rate of CC patients with localized and limited lesion could be as high as 91.5%, while it is only 16.5% in metastatic CC patients.^5^ And approximately 13% of CC patients are diagnosed at advanced clinical stages that characterized by distant metastasis.^6^ Currently, there are several options could be used for the treatment of early‐stage CC patients without metastasis, such as surgical resection, chemotherapy, radiotherapy and the combination of these treatment.^1^ However, there is still no very effective therapy option for metastatic CC patients due to the heterogeneous manifestations.^7^ Therefore, it is essential to explore the underlying mechanisms of CC tumorigenesis for improving the prognosis of CC patients, especially those advanced stages patients.

Circular RNAs (circRNAs) is a novel kind of non‐coding RNAs which differs from the conventional linear RNAs.^8^ It exists widely in mammal cells with a circular structure that has no 5’‐cap and 3’‐poly A tail.^8^ This unique structure gives circRNAs the ability to resist the digestion of RNA exonucleases.^9^ Moreover, its expression profile usually showed cell—tissue—and even time‐specificity under specific conditions.^10^ These characters make it one of the most promising biomarkers for the diagnosis of various human diseases.^11^, ^12^, ^13^ Recently, numerous circRNAs have been identified to be dysregulated during the tumorigenesis of multiple human cancers, such as breast cancer, hepatocellular carcinoma and oral cancer.^14^ The establishment of the whole expression profiles of circRNA in tumour cells must be contributed to the clinical screen of human tumours. Besides, circRNAs have also been demonstrated to play a promotive or suppressive role in the tumour cell proliferation, migration and invasion.^15^ Although the involvement of circRNAs in CC have been reported by several studies,^16^, ^17^ the functions and mechanisms of circRNAs in CC still need further study.

In the present study, we analysed the differentially expressed circRNAs in two CC‐related GSE data sets (GSE113696 and GSE102686), attempting to identify specific circRNAs that involves in the CC tumorigenesis and investigate the underlying mechanism. Our study suggested that circ_0000520 was the only circRNA that dysregulated in both GSE113696 and GSE102686, and qRT‐PCR analysis validated its down‐regulation in CC cell lines. Functional experiments indicated that circ_0000520 served as a CC suppressor in vitro and in vivo. Mechanically, miR‐146b‐3p was predicted and validated to be bind by circ_0000520 and PAX5 mRNA, and circ_0000520 could indirectly modulate PAX5 expression by sponging miR‐146b. In conclusion, circ_0000520 repressed CC progression by indirectly regulating the expression of PAX5 via sponging miR‐146b‐3p.

## 2 MATERIALS AND METHODS

### 2.1 Cervical cancer cell lines

The normal human cervical epithelial cell line (HcerEpic) and cervical cancer cell lines (HeLa, SiHa, SW756, CaSkI and C33A) were brought from type Culture Collection of Chinese Academy of Sciences (Shanghai). All these cell lines were cultured in the foetal bovine (10%, Sigma) contained DMEM (Invitrogen) under 5% CO2 and 95% air at 37°C.

### 2.2 CircRNAs expression profile analysis

Two CC‐related GSE data sets (GSE113696 and GSE102686) were downloaded from the Gene Expression Omnibus database (GEO, http://www.ncbi.nlm.nih.gov/geo), and their circRNAs expression were analysed using GEO2R (http://www.ncbi.nlm.nih.gov/geo/geo2r/).

### 2.3 Quantitative real‐time PCR (RT‐PCR) assay

After exacted the total RNAs from CC cells using TRIzol reagent (Invitrogen), we reversely transcribed 2 μg of total RNAs into cDNA using High‐Capacity cDNA Reverse Transcription Kit (Applied Biosystems). RNaseR treatment was carried out by incubating RNA samples with 2 U RNaseR (Epicenter) for 1 hour at 37°C. RT‐PCR was conducted using BestarTM qPCR MasterMix (DBI Bioscience) on an ABI7500 system. The relative fold‐change was calculated through 2^−ΔΔCt^ method, and the primers were obtained form Invitrogen (Table 1).

**Table 1:** Primer sequences in RT‐PCR assay

Gene	Sequence or target sequence
Circ_0000520‐F	5'‐GGGAAGGTCTGAGACTAGGG‐3'
Circ_0000520‐R	5'‐GGACATGGGAGTGGAGTGAC‐3'
GAPDH‐F	5'‐CACCCACTCCTCCACCTTTG‐3'
GAPDH‐R	5'‐CCACCACCCTGTTGCTGTAG‐3'
PAX5‐F	5'‐ATGGACCAGGGGCCCAAGAGTCCTG‐3'
PAX5‐R	5'‐TCAGTGGCGGTCGTAGGTGGAGGCT‐3'
miR‐146b‐3p‐F	5'‐GACTGCCCTGTGGACTCAGTTC‐3'
miR‐146b‐3p‐R	5'‐GTGCAGGGTCCGAGGTATTC‐3'
U6‐F	5’‐CTCGCTTCGGCAGCACA‐3’
U6‐R	5’‐AACGCTTCACGAATT TGCGT‐3’

### 2.4 MTT assay

Treated HeLa and C33A cells were seeded into 96‐well plates at a density of 4000 cells/well and cultured at 37°C overnight. MTT dye solution (20 μL) was added into each well and cultured for another 4 hours, and then stop solution (200 μL) was added to stop the reaction. Finally, the absorbance was determined with InfiniteVR 200 PRO (Tecan) at 490 nm.

### 2.5 Colony formation assay

Briefly, treated CC cells were seeded into 35‐mm culture dishes at a density of 2000 cells/well. After incubated at 37°C in an incubator with 5% CO_2_ and 95% air for 2 weeks, the colonies were fixed and stained, and the number of colonies was calculated under a microscope.

### 2.6 Cell apoptosis and cycle analysis

Cell apoptosis of treated CC cells was analysed using flow cytometry analysis using Annexin V‐fluorescein isothiocyanate Apoptosis Detection Kit following the instructions. For cell cycle analysis, treated CC cells were digested using trypsin and harvested. After washed twice with PBS, treated CC cells were fixed in 70% ethanol for 2 hours, and then incubated with the mixture of RNase A (50 mg/mL), Triton X‐100 (0.25%) and EDTA (0.1 mmol/L) for 0.5 hours. Next, cells were incubated with 100 μg/mL propidium iodide (PI) for 15 minutes, and the cell cycle was detected by a FACSCalibur instrument (Becton Dickinson) and analysed via Cell Quest software.

### 2.7 Transwell assay

Transwell chambers (Corning Incorporated) with or without Matrigel matrix (BD Biosciences) were used to test the invasive or migratory ability of treated CC cells. In brief, harvested CC cells were re‐suspended in serum‐free DMEM with a concentration of 3 × 10^6^ cells/mL. Then, 200 µL CC cell suspension was added into the upper chamber, and 500 µL FBS (10%) contained DMEM was added into the lower chamber. After 24 hours incubation, the CC cells on the lower compartment were fixed and stained with 0.5% crystal violet (Beyotime Institute of Biotechnology).

### 2.8 Wound‐healing assay

The treated CC cells were seeded into 35 mm culture dishes and allowed to growth to 100% confluence at 37°C. Subsequently, a straight scratch was made in the monolayer using a pipette tip. After culture of 24 hours, the width of scratch was captured and calculated with ImageJ software.

### 2.9 In vivo tumour growth assay

Xenograft tumour model was established in male BALB/c mice (ten weeks old). HeLa cells (2 × 10^8^) stably transfected with circ OE were subcutaneously implanted into the right flank of nude mice. The tumour volume was examined every 7 days since the first day of implantation until 35th. After 35 days, the animals were killed and the tumour were collected and weighted.

### 2.10 RNA immunoprecipitation (RIP) assay

The Magna RIP™RNA Binding Protein Immunoprecipitation Kit (Millipore) was adopted to perform RIP assay following to the protocols of manufacturers. After lysed in RIPA buffer, the cell extracts were subjected for incubation of RIP buffer, which contained magnetic bead conjugated anti‐Ago antibody or IgG. The immunoprecipitated complex was incubated with proteinase K, and then subjected to the detection of qRT‐PCR for the target RNAs.

### 2.11 Western blot

Total proteins were extracted from indicated CC cells using RIPA buffer, and then isolated by 10% SDS‐PAGE. Subsequently, the target proteins were transferred into PVDF membranes followed by the incubation of non‐fat dry milk (5%) for 2 hours. Next, the membranes were probed by primary antibodies that against PAX5 (1:5000, ab109443, Abcam) and β‐catenin (1:5000, #9581, Cell Signaling Technology) for 24 hours, followed by secondary antibody incubation for 2 hours. The bands were visualized by chemiluminescence.

### 2.12 Dual‐luciferase reporter assay

To validate the interplay between circ_0000520 and miR‐146b‐3p, the wild type (WT) and mutant (Mut) sequences of circ_0000520 containing putative miR‐146b‐3p binding sites were sub‐cloned into the pGL3 (Promega) vector to form circ_0000520‐WT and circ_0000520‐Mut recombinant plasmids. HeLa cells and C33A cells were seeded into 96‐well plates at a concentration of 2 × 10^5^ cells/well, and cultured at 37°C overnight. HeLa and C33A cells were co‐transfected with miR‐146b‐3p and circ_0000520‐WT or circ_0000520‐Mut. Then, the firefly and renilla luciferase activities of treated cells were detected using the Dual‐Luciferase Assay System (Promega), and renilla luciferase activity was normalized to Firefly luciferase activity. The interaction between PAX5 and miR‐146b‐3p were also verified as same as miR‐146b‐3p and circ_0000520.

### 2.13 Statistical analysis

Data was presented as mean ± SEM. One‐way analysis of variance, conducted using GraphPad (Ver. Prism 7, GraphPad Prism Software), was applied to analyse the difference between different groups, and P value less than 0.05 was considered significant.

## 3 RESULTS

### 3.1 Circ_0000520 was identified to be decreased in CC

**Figure 1:**
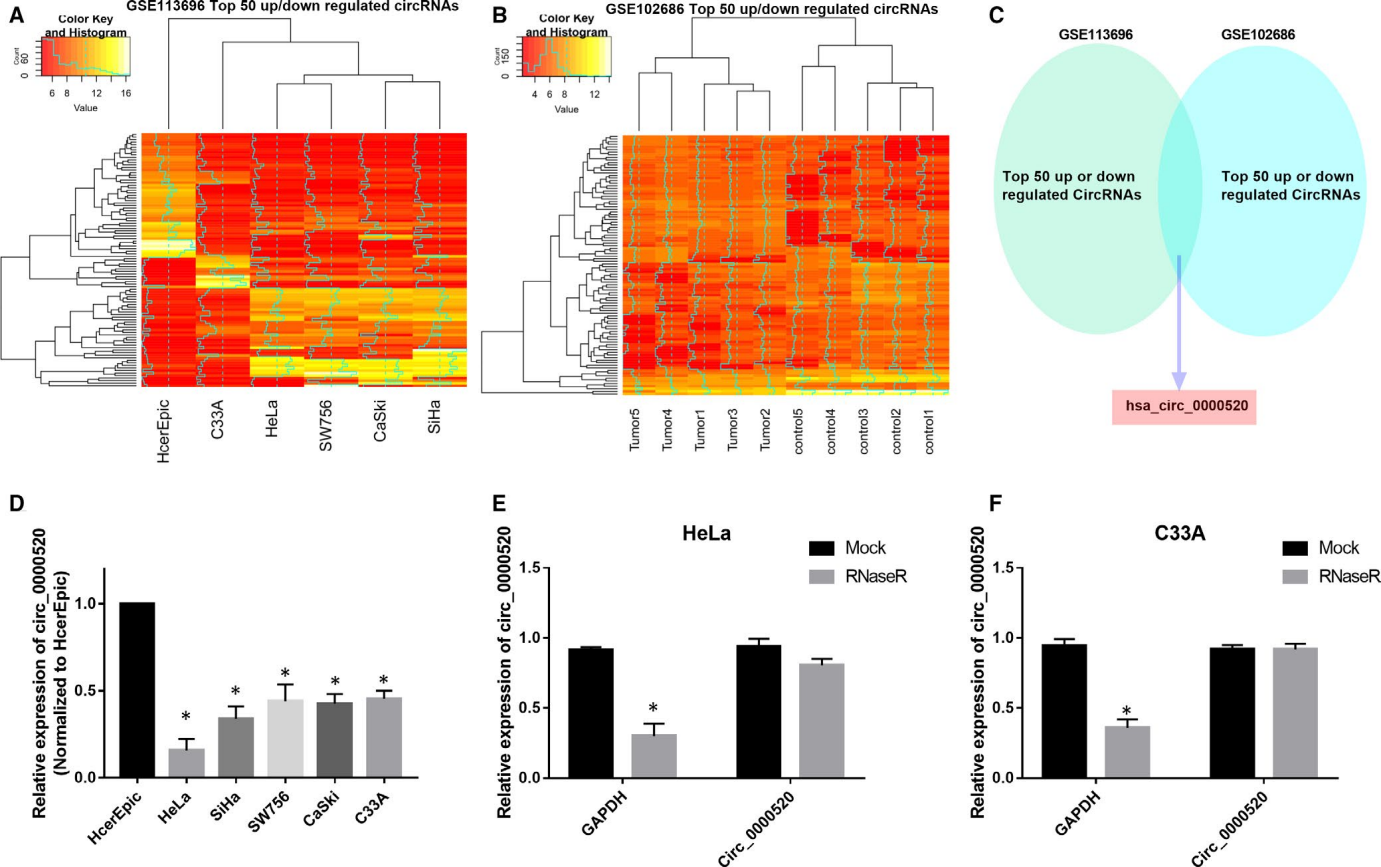
Circ_0000520 was identified to be decreased in CC. A and B, The top 50 dysregulated circRNAs of GSE113696 and GSE102686 data sets were screened by bioinformatics analysis. C, Venn diagram showing the intersection between GSE113696 and GSE102686 data sets. D, Level of circ_0000520 was examined by qRT‐PCR in HcerEpic cell line and five CC cell lines (HeLa, SiHa, SW756, CaSki, and C33A), *P < .05. E and F, The circular structure of circ_0000520 was validated by RNaseR treatment in HeLa and C33A cells, *P < .05

To screen novel CC‐related circRNAs, we performed circRNA microarray analysis in two GSE data sets (GSE113696 and GSE102686). The top 50 dysregulated circRNAs in GSE113696 and GSE102686 were listed (Figure 1A,B). Among these dysregulated circRNAs, circ_0000520 (hsa_circ_0000520, circBase, http://www.circbase.org/) was identified to be the only intersection of GSE113696 and GSE102686 (Figure 1C). Circ_0000520 level was subsequently examined in five CC cell lines, HeLa, SiHa, SW756, CaSki and C33A using qRT‐PCR. Compared to HcerEpic cell line, circ_0000520 was remarkably decreased in HeLa, SiHa, SW756, CaSki and C33A (Figure 1D). To validate the circular structure of circ_0000520, we treated HeLa and C33A cells with RNaseR followed by the detection of circ_0000520 using qRT‐PCR. As results illustrated that RNaseR treatment significantly decreased the expression of GAPDH in HeLa and C33A cells, while it had no effects on the expression level of circ_0000520 (Figure 1E,F). These results indicated circ_0000520 might have a role in the tumorigenesis of CC.

### 3.2 Circ_0000520 overexpression repressed CC cell growth in vitro

**Figure 2:**
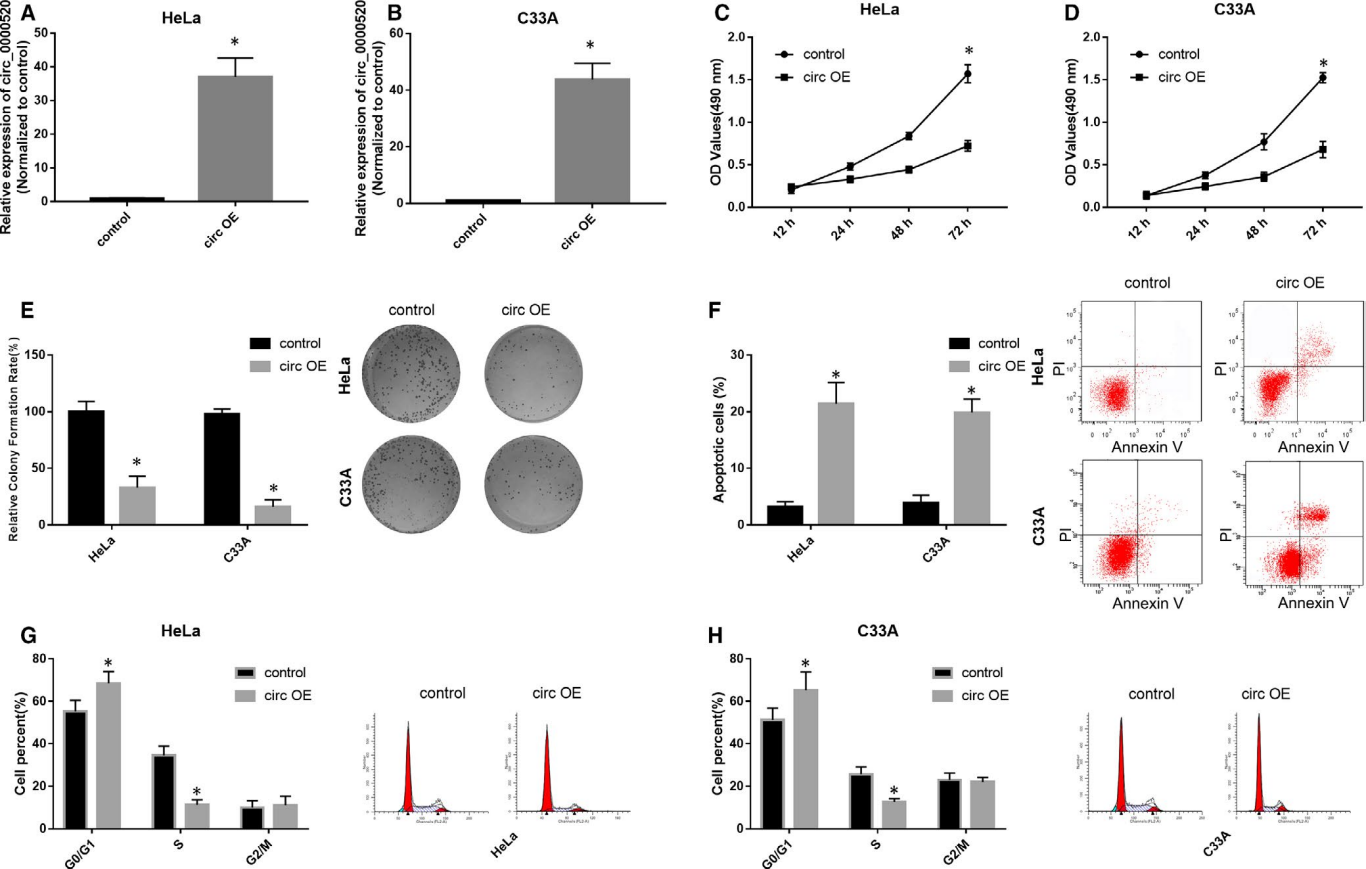
Circ_0000520 overexpression repressed CC cell growth in vitro. A and B, Overexpression efficiency of circ OE in HeLa and C33A cells was determined by qRT‐PCR, *P < .05. C and D, Cell viability of HeLa and C33A cells transfected with control and circ OE plasmid were assessed via MTT assay, *P < .05. E and F, After transfected with circ OE, the cell proliferation and apoptosis of HeLa and C33A cells were analysed by colony formation and flow cytometry, respectively, *P < .05. G and H, Influences of circ OE transfection on HeLa and C33A cell cycle were estimated through flow cytometry analysis, *P < .05

To investigate whether circ_0000520 plays a role in CC tumorigenesis, we overexpressed circ_0000520 in vitro by transfecting HeLa and C33A cells with circ_0000520 overexpression plasmid (circ OE), subsequently, analysed the effects of circ_0000520 overexpression on CC cell proliferation, apoptosis and cell cycle. Firstly, we measured the overexpression efficiency of circ OE in HeLa and C33A cells by qRT‐PCR. Results showed that circ_0000520 level was markedly increased in circ OE transfected HeLa and C33A cells compared to those control cells **(**Figure 2A,B), indicating that circ OE treatment significantly overexpressed circ_0000520 in HeLa and C33A cells. Subsequently, we demonstrated that circ_0000520 overexpression remarkably reduced the cell viability of HeLa and C33A cells through MTT assay (Figure 2C,D). Circ_0000520 overexpression was also found to repress the cell proliferation of HeLa and C33A cells in colony formation assay (Figure 2E). Moreover, in the apoptosis, we found that circ_0000520 overexpression resulted in a remarkable up‐regulation of cell apoptosis rate in HeLa and C33A cells (Figure 2F). In addition, in the cell cycle analysis, the G0/G1 phases cell number of HeLa and C33A cells transfected with circ OE were significantly increased compared to control RNA transfected cells; while the S phase cell number of HeLa and C33A cells transfected with circ OE were remarkably reduced (Figure 2G,H). These findings suggested that circ_0000520 overexpression repressed CC cell growth in vitro.

### 3.3 Circ_0000520 overexpression repressed CC cell migration and invasion in vitro

**Figure 3:**
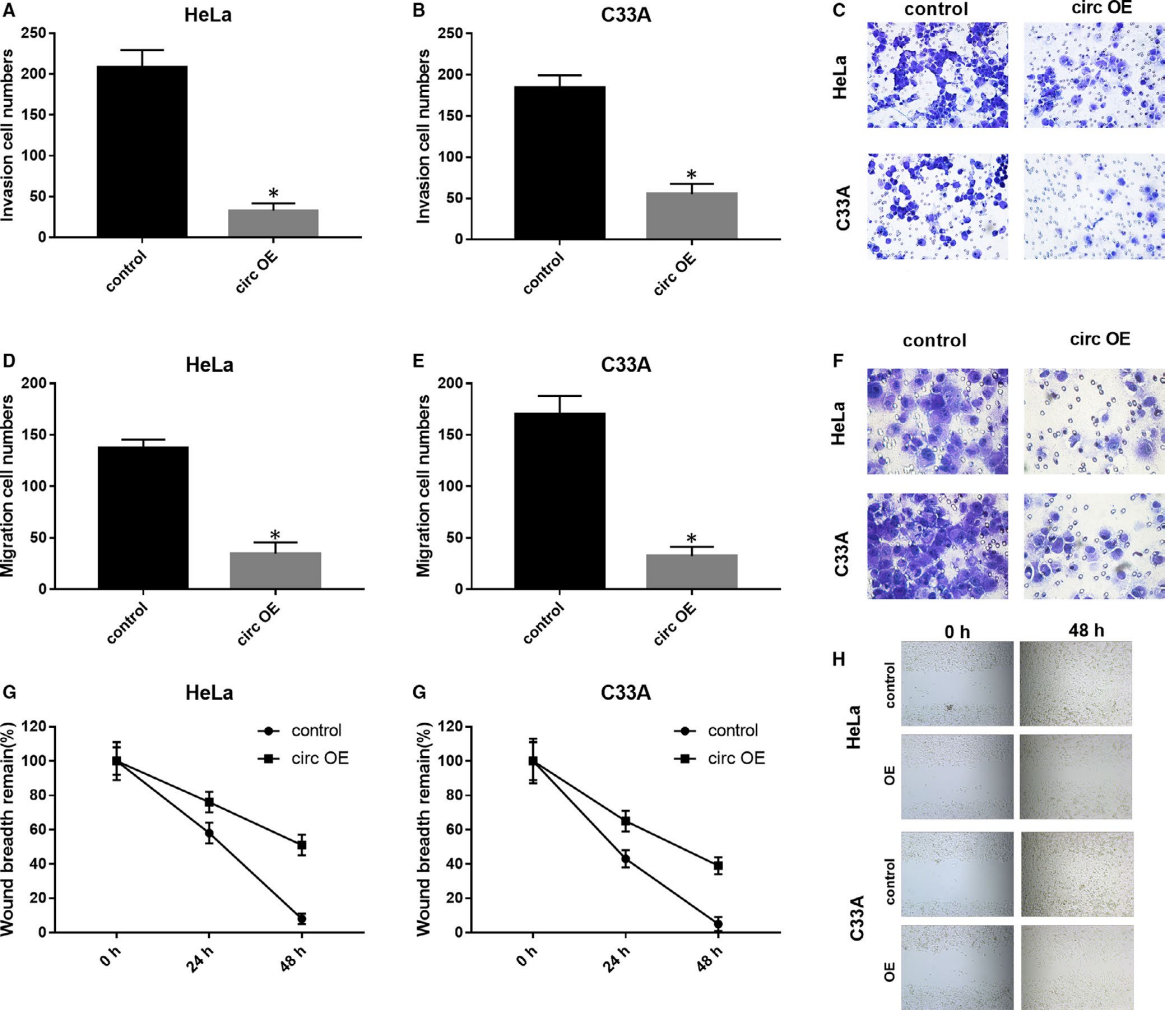
Circ_0000520 overexpression repressed CC cell migration and invasion in vitro. A‐C, Chambers coated with Matrigel matrix were used in transwell assay to estimate the effects of circ_0000520 on CC cell invasion, *P < .05. D‐F, Chambers without Matrigel matrix were used in transwell assay to examine the circ_0000520 overexpressed HeLa and C33A cell migratory capacity, *P < .05. G‐H, Wound‐healing experiment carried out in HeLa and C33A cell was utilized to test the effects of circ_0000520 OE on cell migration

We further evaluated the effects of circ_0000520 overexpression on the migratory and invasive capacities of CC cell in vitro via transwell and wound‐healing experiments. According to the results from transwell assay using Matrigel matrix coated chamber, circ OE transfection reduced the invaded cell number of HeLa and C33A cells compared to control RNA transfection (Figure 3A‐C). Moreover, by using chambers without Matrigel matrix, we demonstrated that circ_0000520 overexpression also reduced the migration cell number of HeLa and C33A cells (Figure 3D‐F). In addition, wound‐healing assay was performed in HeLa and C33A cells to further assess the influences of circ_0000520 overexpression on cell migration. As results indicated that circ_000052 overexpression of HeLa and C33A cells remarkably slowed the healing of wounds (Figure 3G‐I). Taken together, circ‐0000520 overexpression was demonstrated to repress CC cell migration and invasion.

### 3.4 Circ_0000520 overexpression repressed CC progression in vivo

**Figure 4:**
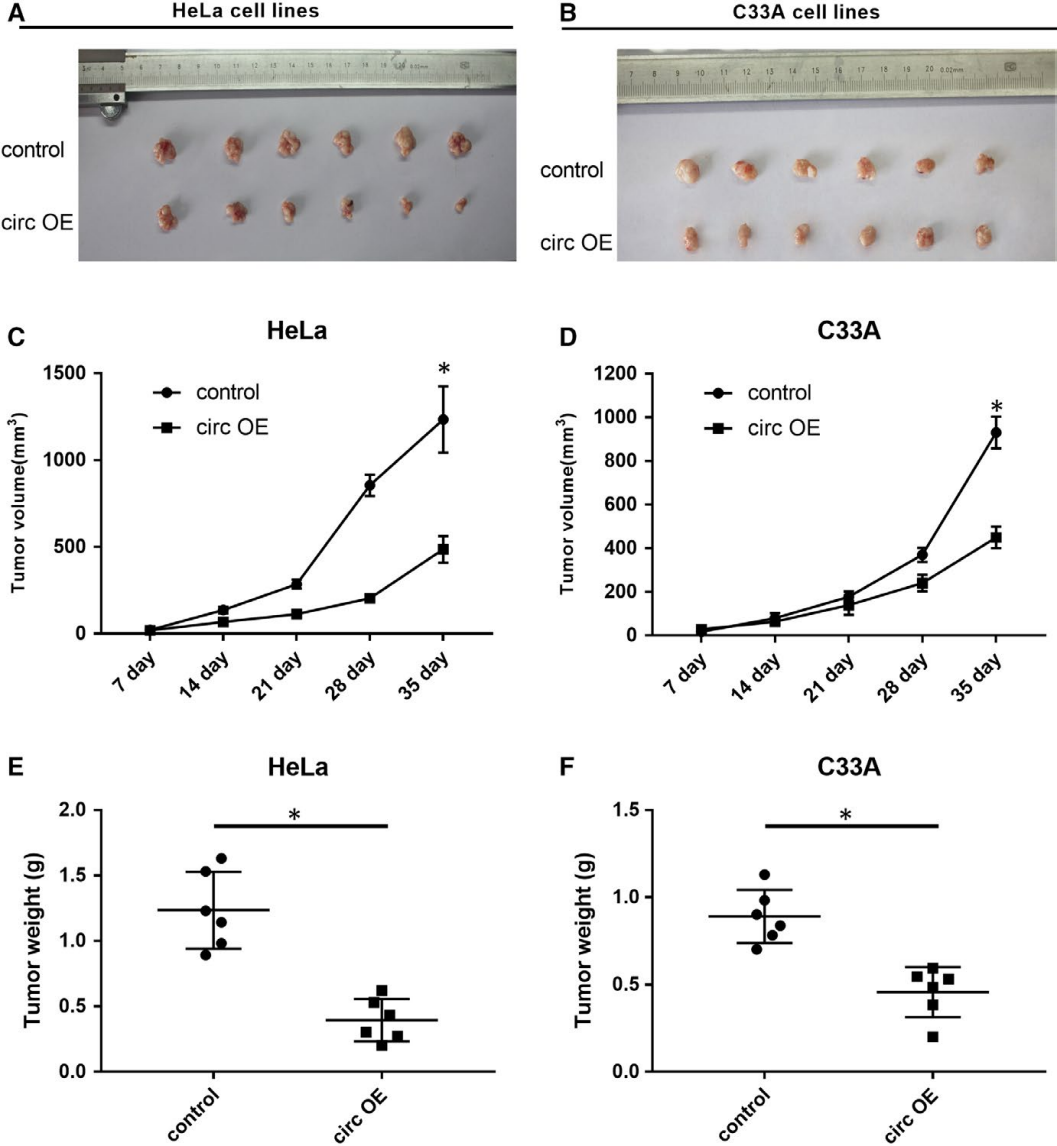
Circ_0000520 overexpression repressed CC progression in vivo. HeLa and C33A cells stably transfected with circ OE were subcutaneously injected into nude mice, and then the tumour volume and weight were assessed. A and B, Representative diagrams of xenograft tumours formed by circ OE treated HeLa and C33A cells. C and D, The volume of xenograft tumours of HeLa and C33A groups were measured every 7 days since the first day of transfection until 35th, *P < .05. E and F, After 35 days of inoculation, xenograft tumours were resected and weighted, *P < .05

In vivo xenograft assay was performed to further confirm the suppressive functions in CC cell growth. HeLa and C33A cells stably transfected with circ OE were subcutaneously injected into the left flank of nude mice, the tumour growth was examined every 7 days since the first day of injection until 35th. The representative diagrams indicated that the tumours from circ OE transfected group were smaller than those from control group (Figure 4A,B). Growth curve of tumour showed that circ_0000520 overexpression significantly slowed the CC tumour growth (Figure 4C,D). Finally, the animals were killed and the tumours were resected and weighted. Compared to the tumours form control group, the weight of tumours from circ OE group was significantly reduced (Figure 4E,F). These results further supported the repressive role of circ_0000520 in CC in vivo.

### 3.5 Circ_0000520 acted as a sponge of miR‐146b‐3p in CC

**Figure 5:**
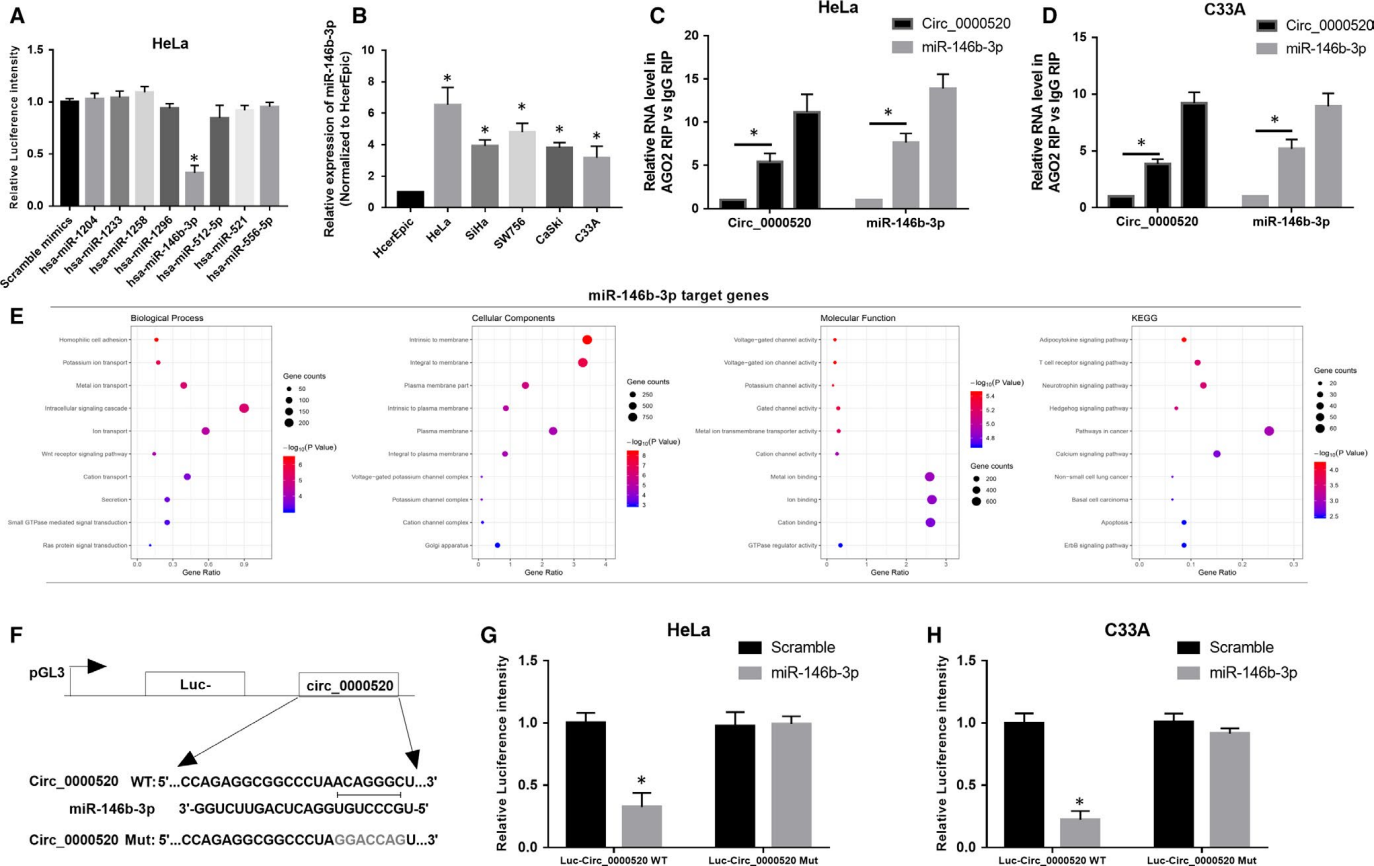
Circ_0000520 acted as a sponge of miR‐146b‐3p in CC. A, Nine target miRNAs of circ_0000520 predicted by CirRNA interactome were validated by dual‐luciferase reported assay in HeLa cells, *P < .05. B, Relative expression level of miR‐146b‐3p in HcerEpic cell line and five CC cell lines (HeLa, SiHa, SW756, CaSki, and C33A) were examined by qRT‐PCR, *P < .05. C and D, Anti‐AGO2 RIP experiment was performed in HeLa and C33A cells to validate the interaction between circ_0000520 and miR‐146b‐3p, *P < .05. E, KEGG analysis of miR‐146b‐3p. F, The schematic diagram of construction of circ_0000520 reported plasmid. G and H, Dual‐luciferase reported experiment was performed in HeLa and C33A cells co‐transfected with Luc‐Circ_0000520 WT or Luc‐Circ_0000520 Mut and miR‐146b‐3p or scramble control RNA to validate the interplay between circ_0000520 and miR‐146b‐3p, *P < .05

Considering the repressive roles of circ_0000520 were demonstrated in vitro and in vivo, we then wondered how it works during CC tumorigenesis. Due to numerous circRNAs have been identified to play a role in tumour progression through acting as sponges of miRNAs, we focused our research on screening the potential target miRNAs of circ_0000520. A total of nine miRNAs were identified by CirRNA interactome (https://circinteractome.nia.nih.gov/index.html) to be the potential targets of circ_0000520, and luciferase reporter assay was performed to validate the interplay between the nine miRNAs and circ_0000520 in HeLa cells. As results indicated that only miR‐146b‐3p could attenuate the luciferase activity of HeLa cells driven by circ_0000520 (Figure 5A). Results from qRT‐PCR suggested that miR‐146b‐3p expression in five CC cell lines (HeLa, SiHa, SW756, CaSki and C33A cell lines) was significantly higher than that in HcerEpic cell line (Figure 5B). We adopted anti‐AGO2 immunoprecipitation (RIP) experiment to validate the interplay between circ_0000520 and miR‐146b‐3p in HeLa and C33A cells. As results indicated that circ_0000520 and miR‐146b‐3p were mainly enriched in anti‐Ago treated HeLa and C33A cells (Figure 5C,D), indicating that circ_0000520 is recruited to an Ago‐related complex where by it interacts with miR‐146b‐3p. GO analysis was adopted to detect the enrichments of miR‐146b‐3p target gene in terms of biological process (BP), cellular component (CC) and molecular function (MF). The top term under BP, CC and MF are ‘Intracellular signaling cascade’, ‘Intrinsic to membrane’ and ‘Metal ion binding’, respectively (Figure 5E). Moreover, KEGG analysis revealed the most relevant pathway is “Pathways in cancer” (Figure 5E). In addition, dual‐luciferase reporter experiment was conducted in HeLa and C33A cells to confirm the interplay between circ_0000520 and miR‐146b‐3p. The luciferase activity of HeLa and C33A cells driven by Luc‐Circ_0000520 WT could only be attenuated by the transfection of miR‐146b‐3p, while the luciferase driven by Luc‐Circ_0000520 WT was not affected by miR‐146b‐3p (Figure 5F‐H). These results showed that circ_0000520 acted as a sponge of miR‐146b‐3p in CC.

### 3.6 miR‐146b‐3p targeted and negatively regulated PAX5 in CC

**Figure 6:**
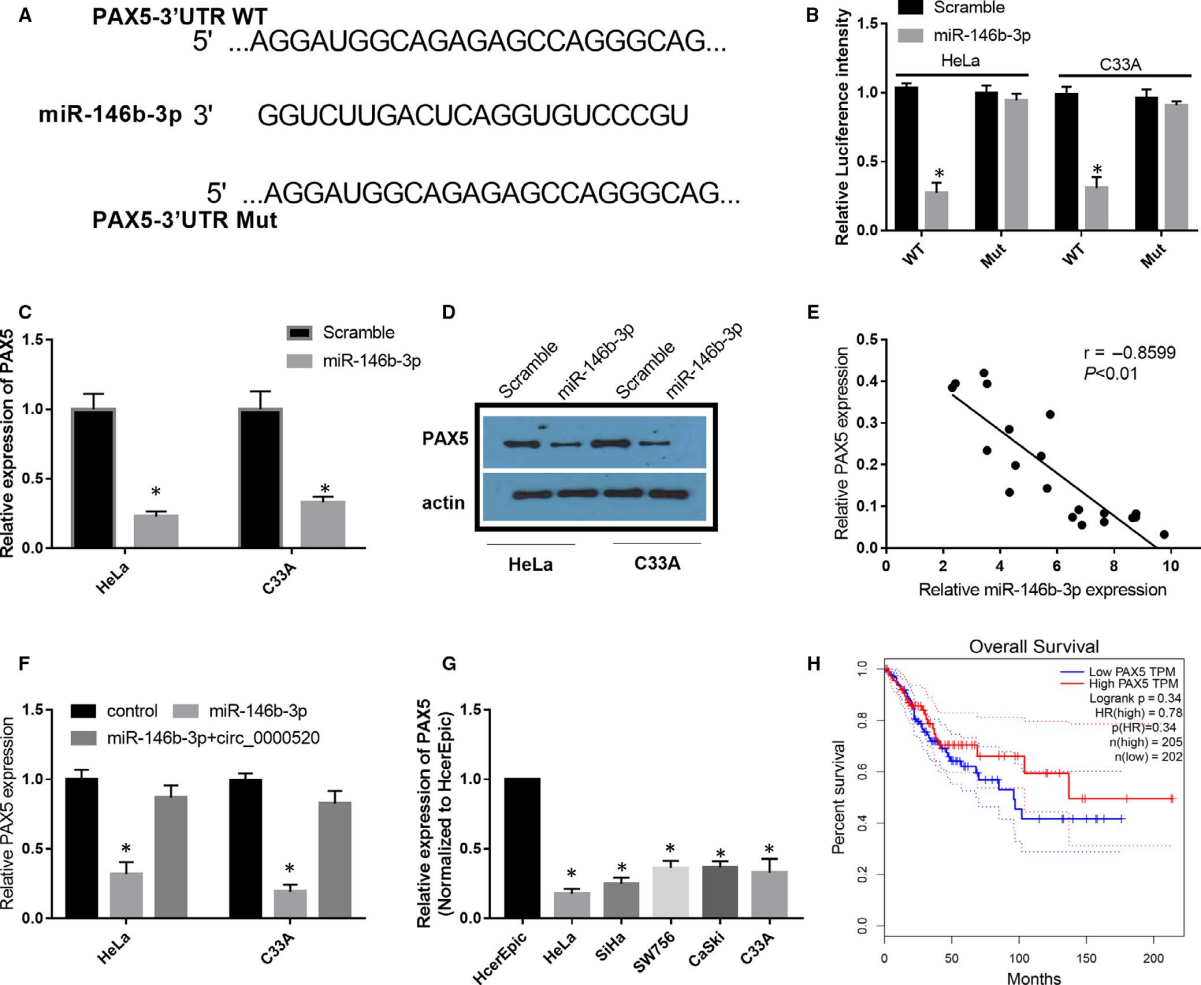
miR‐146b‐3p targeted and negatively regulated PAX5 in CC. A, The sequences of putative binding sites between miR‐146b‐3p and PAX5 mRNA. B, Dual‐luciferase reported experiment was performed in HeLa and C33A cells co‐transfected with PAX5 WT or PAX5 Mut and miR‐146b‐3p or scramble control RNA to validate the interplay between PAX5 and miR‐146b‐3p, *P < .05. C and D, PAX5 mRNA and protein levels in HeLa and C33A cells transfected with miR‐146b‐3p and scramble control were detected via qRT‐PCR and western blot assays, *P < .05. E, Correlation between the expression of PAX5 and miR‐146b‐3p in CC samples were analysed by Pearson's correlation method (r=−0.8599, *P* < .01). F, PAX5 mRNA level was examined in control, miR‐146b‐3p treated, and miR‐146b‐3p + circ_0000520 treated HeLa and C33A cells with qRT‐PCR, *P < .05. G, Relative PAX5 level in HcerEpic cell line and five CC cell lines (HeLa, SiHa, SW756, CaSki, and C33A) were assessed, *P < .05. H, The overall survival rate of patients with low and high PAX5 was analysed

To further explore how miR‐146b‐3p involves in the suppressive effects of circ_0000520 on the tumorigenesis of CC, we screened the target genes of miR‐146b‐3p by bioinformatics measures. Results from prediction showed that the 3’‐UTR of PAX5 mRNA possessed the miR‐146b‐3p complementary sequences (Figure 6A). In the dual‐luciferase reporter assay, we found that miR‐146b‐3p treatment significantly reduced the luciferase intensity of HeLa and C33A cells driven by PAX5‐WT, but not PAX5‐Mut (Figure 6B). While the luciferase activity of HeLa and C33A cells driven by PAX5‐Mut was not affected by miR‐146b‐3p (Figure 6B). We then found that the mRNA and protein expression of PAX5 were significantly decreased in miR‐146b‐3p transfected HeLa and C33A cells compared to those scramble control RNA transfected cells (Figure 6C,D). Moreover, a negative correlation was observed between the expression levels of PAX5 and miR‐146b‐3p in CC samples (Figure 6E). To assess the regulatory net between circ_0000520, miR‐146b‐3p and PAX5, we detected PAX5 level in HeLa and C33A cells transfected with miR‐146b‐3p and miR‐146b + circ_0000520 by qRT‐PCR. As results indicated that miR‐146b‐3p overexpression resulted in a significant down‐regulation of PAX5 in HeLa and C33A cells, while co‐transfection of miR‐146b‐3p and circ_0000520 abrogated this miR‐146b‐3p induced down‐regulation of PAX5 (Figure 6F). Compared to HcerEpic cell line, PAX5 was remarkably decreased in HeLa, SiHa, SW756, CaSki and C33A cells (Figure 6G). In addition, we found that patients with high PAX5 expression showed a better overall survival rate than those patients with low PAX5 expression (Figure 6H). Taken together, circ_0000520 might indirectly release PAX5 from miR‐146b‐3p by sponging to miR‐146b‐3p.

### 3.7 Knockdown of PAX5 abolished the inhibitory effects of circ_0000520 on CC cell proliferation and invasion

**Figure 7:**
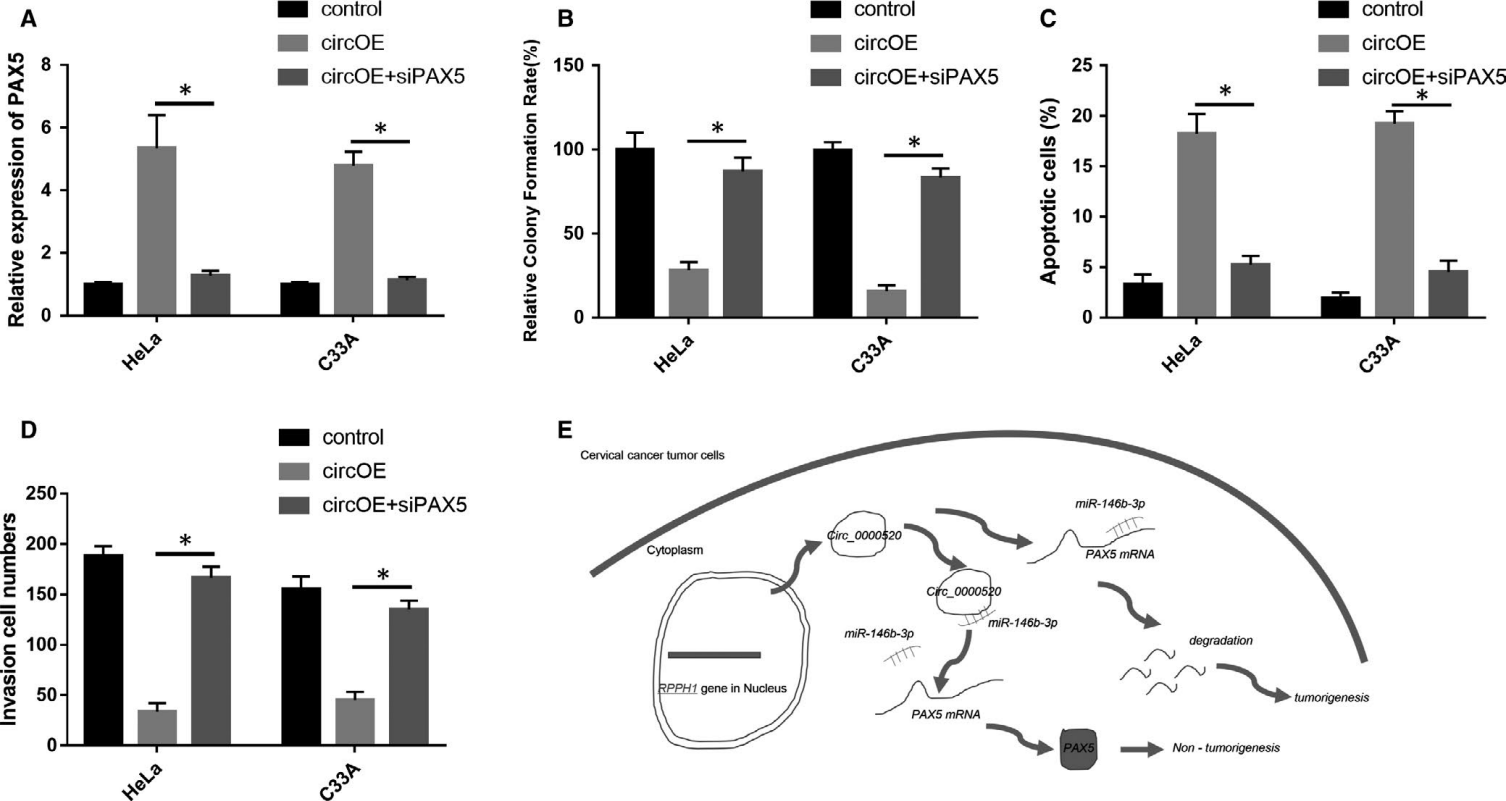
Knockdown of PAX5 abolished the inhibitory effects of circ_0000520 on CC cell proliferation and invasion. A, PAX5 was examined in circ OE or circ OE+siPAX5 transfected HeLa and C33A cells using qRT‐PCR, *P < .05. B, Colony formation, C, flow cytometry and D, transwell assays were performed to investigate the effects of siPAX5 transfection on the cell proliferation, apoptosis and invasion, respectively, in circ_0000520 overexpressed HeLa and C33A cells, *P < .05. E, The diagram of the molecular mechanisms underlying the circ_0000520/miR‐146b‐3p/PAX5 axis in CC

The exist of circ_0000520/miR‐146b‐3p/PAX5 axis was demonstrated in above, we then attempted to further investigate its function in CC cell growth in vitro. We found that PAX5 was significantly increased when transfected HeLa and C33A cells with circ OE, while co‐transfected HeLa and C33A cells with circ OE and siPAX5 abolished the circ OE induced up‐regulation of PAX5 (Figure 7A). We then examined whether siPAX5 could rescue the inhibitory effects of circ OE on HeLa and C33A cell proliferation and invasion. Results from colony formation assay suggested that co‐transfected HeLa and C33A cells with circ OE and siPAX5 reversed the circ OE induced inhibition of cell proliferation (Figure 7B). Co‐transfection of circ OE and siPAX5 also abolished the circ OE caused promotion of cell apoptosis in HeLa and C33A cells (Figure 7C). In the invasion analysis, we demonstrated that co‐transfected HeLa and C33A cells with circ OE and siPAX5 abrogated the circ OE induced suppression of cell invasion (Figure 7D). In conclusion, circ_0000520 promoted CC tumorigenesis by acting as a sponge of miR‐146b‐3p to release PAX5 expression.

## 4 DISCUSSION

Currently, CC ranks as the fourth most common tumour in women throughout the whole world.^18^ There are several risk factors for CC, of which HPV infection is the major cause of CC initiation.^19^ CircRNAs have long been considered to be molecular flukes since its first discovery in 1970s by Sanger HL.^20^ Fortunately, with the tremendous progress of next‐generation sequencing technology and bioinformatics analysis, the biological functions of circRNAs in mammals have been well recognized.^9^, ^10^ Abnormal expression prolife of circRNAs were frequently found in the specific area of tumours, implying a critical role of circRNAs during the tumorigenesis.^21^ Indeed, increasing evidences have shown the significant roles of circRNAs in human cancer progression.^21^ Recently, multiple circRNAs were revealed to affect almost all the aspects of CC tumour development, including cell proliferation, invasion and migration.^22^, ^23^ For instance, Tian and Liang reported that circSMARCA5 was lowly expressed in CC, and its overexpression caused a significant repression on CC cell proliferation, invasion and migration, as well as induced a cell cycle arrest.^24^ Moreover, circ_0018289 was screened as the most prominent one among the 45 dysregulated circRNAs that identified by microarray analysis in CC tissues.^25^ In the further study, the author demonstrated that circ_0018289 functioned as an oncogene of CC through sponging miR‐497.^25^ Circ_0000520 was previously identified by Sun H et al to be remarkably reduced in gastric cancer tissues, and its level was negatively correlated to the tumour node metastasis(TNM) stages.^26^ However, they did not explore the biological functions of circ_0000520 in the tumorigenesis of gastric cancer. To our best knowledge, the function of circ_0000520 in human cancers has not been investigated yet. In this study, we conducted a circRNA microarray analysis in two CC‐related GSE data sets and revealed that circ_0000520 is the only intersection of the top 50 dysregulated circRNAs of GSE113696 and GSE102686. By silencing its expression in vitro and in vivo, we demonstrated that circ_0000520 is a tumour repressor of CC. These findings implied that circ_0000520 is a promising RNA molecule for CC screen and therapy.

Up to now, studies have revealed that circRNAs could participate in the tumour progression through multiple distinct mechanisms, of which miRNA sponging is the most common one of them.^16^, ^27^ MiRNAs is a subtype of non‐coding RNAs with short nucleotides that could post‐transcriptionally regulate the expression of target mRNAs through miRNAs response elements (MREs).^28^ A considerable portion of circRNAs possess MREs, indicating that they could reduce the binding of miRNA to its target mRNAs, thereby indirectly release the miRNAs targets. The role of circRNAs in modulating tumour‐related genes via fine‐tuning miRNAs has been widely accepted. Increasing number of circRNAs were identified as miRNA sponges in various cancers,^29^, ^30^ however, this mechanism revealed in CC is very limited. In this study, circ_0000520 was identified as a sponge of miR‐146b‐3p.

After having validated the interplay between circ_0000520 and miR‐146b‐3p, we further predicted the downstream genes that may contribute to the functional role of circ_0000520. PAX5 was revealed to be the direct target gene of miR‐146b‐3p and it could be regulated by circ_0000520. Moreover, the rescue assays suggest that circ_0000520 regulated CC cell proliferation and invasion through sponging miR‐146b‐3p and subsequently releasing PAX5.

## 5 CONCLUSIONS

Our study analysed the circRNAs expression profile of CC and characterized circ_0000520 as a research object. Our findings indicated that circ_0000520 may serve as a novel CC repressor through sponging miR‐146b‐3p and thereby releasing PAX5, suggesting that circ_0000520 is a new promising diagnostic and therapeutic RNA molecule of CC.

## CONFLICT OF INTEREST

The authors have no commercial or other associations that might pose a conflict of interest.

## AUTHORS’ CONTRIBUTIONS

Zhang JL and Cai RY performed most of the experiments, interpreted the results and wrote the paper. Zhang YF helped to performed bioinformatics analysis. Zhang JL and Cai RY participated in vitro and in experiments. Zhang JL, Cai RY and Zhang YF write the paper, revised the paper, supervised and approved the study. All authors read and approved the final manuscript.

## Data Availability

The current study is available from the corresponding author upon reasonable request.
